# Soil microbial community response to ectomycorrhizal dominance in diverse neotropical montane forests

**DOI:** 10.1007/s00572-023-01134-4

**Published:** 2024-01-06

**Authors:** Joseph D. Edwards, Alexander H. Krichels, Georgia S. Seyfried, James Dalling, Angela D. Kent, Wendy H. Yang

**Affiliations:** 1 Department of Ecology and Evolutionary Biology, University of Tennessee, Knoxville, TN USA; 2 USDA Forest Service, Rocky Mountain Research Station, Albuquerque, NM 87102 USA; 3 Department of Forest Ecology and Resource Management, Oregon State University, Corvallis, OR 97331 USA; 4 Department of Plant Biology, University of Illinois at Urbana-Champaign, Urbana, IL 61801 USA; 5 Department of Natural Resources and Environmental Science, University of Illinois at Urbana-Champaign, Urbana, IL 61801 USA

**Keywords:** Tropical forest, Mycorrhizal associations, Microbial community, Fungal pathogens, Mycorrhizal-bacterial interactions

## Abstract

Ectomycorrhizal (EM) associations can promote the dominance of tree species in otherwise diverse tropical forests. These EM associations between trees and their fungal mutualists have important consequences for soil organic matter cycling, yet the influence of these EM-associated effects on surrounding microbial communities is not well known, particularly in neotropical forests. We examined fungal and prokaryotic community composition in surface soil samples from mixed arbuscular mycorrhizal (AM) and ectomycorrhizal (EM) stands as well as stands dominated by EM-associated *Oreomunnea mexicana* (Juglandaceae) in four watersheds differing in soil fertility in the Fortuna Forest Reserve, Panama. We hypothesized that EM-dominated stands would support distinct microbial community assemblages relative to the mixed AM-EM stands due to differences in carbon and nitrogen cycling associated with the dominance of EM trees. We expected that this microbiome selection in EM-dominated stands would lead to lower overall microbial community diversity and turnover, with tighter correspondence between general fungal and prokaryotic communities. We measured fungal and prokaryotic community composition via high-throughput Illumina sequencing of the *ITS2* (fungi) and *16S* rRNA (prokaryotic) gene regions. We analyzed differences in alpha and beta diversity between forest stands associated with different mycorrhizal types, as well as the relative abundance of fungal functional groups and various microbial taxa. We found that fungal and prokaryotic community composition differed based on stand mycorrhizal type. There was lower prokaryotic diversity and lower relative abundance of fungal saprotrophs and pathogens in EM-dominated than AM-EM mixed stands. However, contrary to our prediction, there was lower homogeneity for fungal communities in EM-dominated stands compared to mixed AM-EM stands. Overall, we demonstrate that EM-dominated tropical forest stands have distinct soil microbiomes relative to surrounding diverse forests, suggesting that EM fungi may filter microbial functional groups in ways that could potentially influence plant performance or ecosystem function.

**Supplementary Information:**

The online version contains supplementary material available at 10.1007/s00572-023-01134-4.

## Introduction

Plants often influence surrounding soils via interactions with microbial organisms (Zak et al. 2003). Predominant among these plant–microbe interactions are mycorrhizal associations between fungi and plant roots (Hawkes et al. 2007). Two main types of mycorrhizal association, namely, arbuscular mycorrhizal (AM) and ectomycorrhizal (EM), can impact plant health (Revillini et al. 2016), litter decomposition (Jacobs et al. 2018), nutrient cycling (Phillips et al. 2013), and soil organic matter dynamics (Frey 2019). Importantly, mycorrhizal fungi facilitate plant acquisition of soil nutrients such as nitrogen and phosphorous to support plant growth and nutrition (Smith and Smith 2011). However, bacteria, archaea, and non-mycorrhizal fungi also contribute to mycorrhizal plant and ecosystem effects as they can influence nutrient transformation processes, promote plant root exudation, and benefit plant growth (Tarkka et al. 2018; Sangwan and Prasanna 2022; Berrios et al. 2023). These non-mycorrhizal microbial communities are likely responsible for the extended plant-soil effects of mycorrhizal associations that can influence overall forest population dynamics in temperate ecosystems (Bennett et al. 2017). However, we know very little about how these “mycorrhizosphere” (Rambelli 1973) microbiomes manifest in tropical ecosystems that have differing patterns of productivity, species diversity, and soil functionality (Barlow et al. 2018).

Neotropical forests are generally a matrix of primarily AM-associating tree species interspersed with fewer EM-associated species (McGuire et al. 2012), but EM-associated species can also form stands where the majority of the total basal area comprises a single species (Hart et al. 1989; Peh et al. 2011). These EM-dominated stands often differ significantly from surrounding mixed-mycorrhizal forest stands in important ecosystem characteristics (Torti et al. 2001), with slower decomposition rates (McGuire et al. 2010) and lower soil inorganic nutrient availability (Corrales et al. 2016b) than adjacent mixed AM-EM forest stands. Ectomycorrhizal-dominated stands in these forests are also associated with changes to the relative abundance of some fungal functional guilds (Seyfried et al. 2022), but the impact of EM dominance on the broader complex soil microbiome, particularly prokaryotic communities, is still unclear due to a relative lack of data from neotropical forests.

Mycorrhizal fungi often interact with surrounding microbial communities in ways that create favorable ecological conditions for their plant partners (Uroz et al. 2019). These interactions may play an important role in the formation or proliferation of EM-dominated forest stands. For example, EM fungi are thought to outcompete saprotrophic organisms for nutrients in soil organic matter (SOM; Averill and Hawkes 2016) due to their ability to produce SOM-degrading extracellular enzymes (Pellitier and Zak 2018). This fungal interguild competition can increase soil carbon (C)-to-nutrient ratios, slowing C and inorganic nutrient cycling (Fernandez and Kennedy 2016). These changes could decrease overall microbial C and nutrient availability, potentially resulting in lower overall microbial diversity with increasing EM dominance (Eagar et al. 2021; Heděnec et al. 2023), as well as downstream impacts on copiotrophic or oligotrophic soil microbiota (Nemergut et al. 2010). Ectomycorrhizal-associated changes to non-mycorrhizal microbial communities associated with soil organic matter cycling could potentially further promote positive feedbacks to slow SOM cycling and create a competitive advantage for EM mutualisms. Arbuscular mycorrhizal fungi are often thought to scavenge inorganic nutrients from soil and must rely on surrounding microbial communities to degrade SOM (van Der Heijden et al. 2015). Mycorrhizal fungi also influence the activity and abundance of soil fungal pathogens (Borowicz 2001; Veresoglou and Rillig 2012). Both AM and EM fungi can confer pathogen resistance to their plant hosts through the release of volatile organic compounds (Dreischhoff et al. 2020) or extracellular secretion of secondary metabolites (Pellegrin et al. 2015). However, EM relationships can generate greater conspecific benefits for pathogen suppression than AM, potentially promoting EM dominance (Liang et al. 2020) and resulting in greater conmycorrhizal plant recruitment (Delavaux et al. 2023). Overall, the effects of mycorrhizal associations on surrounding microbial community function are highly context-dependent, with their outcome varying based on mycorrhizal fungal species (Emmett et al. 2021), plant species and litter quality (Fernandez et al. 2019), climate (Bennett and Classen 2020), and soil parent material (Seyfried et al. 2021b). Quantifying the effects of EM dominance on the different constituents of the soil microbiome could provide valuable insight to help contextualize the effects of mycorrhizal associations on surrounding soil microbiomes.

To characterize soil microbiome responses to EM tree dominance in a neotropical forest, we conducted a study comparing bulk soil fungal and prokaryotic communities between EM-dominated (by *Oreomunnea mexicana* at > 50% basal area per stand) and mixed AM-EM stands of highly diverse lower montane tropical forests in western Panama. While these sites also contain ~ 14 other EM tree species, those species occur in low abundance, and *O*. *mexicana* is the only one to form monodominant stands (Prada et al. 2017). Variation in parent material and geology among these stands leads to the formation of soils differing in nutrient availability, pH, and base saturation (Prada et al. 2017; Seyfried et al. 2021a), allowing for the investigation of microbial relationships with stand mycorrhizal type across a range of soil fertilities. We hypothesized that EM-dominated stands would be associated with distinct fungal and prokaryotic community assemblages relative to the AM-EM mixed stands. Given that EM dominance can slow SOM cycling and reduce nutrient availability, we present three predictions related to microbiome differences between stand mycorrhizal types (1) that EM stands would be associated with decreased microbial community diversity and heterogeneity compared to mixed AM-EM stands; (2) that EM stands would be associated with decreased relative abundance of saprotrophic and pathogenic fungal functional guilds, as well as bacteria and archaea associated with SOM cycling, compared to AM-EM mixed stands; and (3) that microbial communities in EM-dominated stands would have tighter Procrustean correspondence between general fungal and prokaryotic communities, as EM fungi could be acting to filter the soil microbiome to select microbial organisms that may contribute to an EM competitive advantage.

## Methods

### Study design

This study was conducted in four adjacent watersheds in the Fortuna Forest Reserve of western Panama: Alto Frio, Honda, Hornito, and Pinola/Zorro. We chose to use Pinola/Zorro as a paired site (collectively referred to hereafter as “Pinola”) because there were no EM-dominated stands at the Pinola site and the Zorro site was the closest alternative (~ 800 m away, although on differing parent material). These diverse neotropical montane forests differ in elevation, parent material, soil chemistry, and tree species diversity, containing between 61 and 153 (primarily AM-associating) species/ha (Prada et al. 2017), but also large (> 1 ha) patches where > 50% of the basal area is made up of the EM-associated species *O*. *mexicana* (Table 1; Dalling and Turner 2021). In April 2015, three soil samples were collected from one EM-dominated and from one mixed AM-EM stand within each watershed (4 watersheds × 2 stand mycorrhizal stand types × 3 replicates = 24 samples in total). The soil samples were collected from 0–10 cm depth using a 10 cm diameter soil corer after removing the leaf litter layer; thus, ~ 785 cm^3^ of soil was collected for each sample. Samples contained soil from both organic and mineral horizons, as we did not separate these layers due to high variability in organic horizon depth. Within each stand, the three soil samples collected in each stand were from locations separated from one another by ~ 5 m, and all samples were collected > 1 m away from the nearest tree trunk to limit localized effects of individual species on microbial community composition. Soil samples were stored in insulated coolers with ice packs and transported to the Smithsonian Tropical Research Institute’s Naos Marine and Molecular Laboratories in Panama within 2 days of collecting. At Naos, samples were passed through a 2-mm sieve (sterilized with 70% EtOH between samples), and roots/course organic matter was removed. After processing, samples were stored at − 80 °C for up to 1 week prior to DNA extraction.

**Table 1:** Site characteristics for the four watersheds in the Fortuna Forest Reserve, Panama, that were included in this study

	Alto Frio	Honda	Hornito	Pinola (AM-EM mixed only)	Zorro (EM dominated only)
Lat/Long	8.654, − 82.215	8.751, − 82.239	8.674, − 82.214	8.754, − 82.259	8.761, − 82.261
Elevation (m)	1100	1155	1330	1135	1249
Parent material	Undifferentiated volcanics	Rhyolite	Dacite	Basalt	Granodiorite
Annual precipitation (mm)	4641 (632)	6255 (962)	5164 (232)	4964 (863)	-
Bulk density (g cm^−3^)	0.66 (0.02)	0.29 (0.02)	0.26 (0.03)	0.50 (0.02)	-
pH	5.62 (0.22)	3.58 (0.21)	5.03 (0.68)	5.44 (0.19)	-
Total C (mg cm^−3^)	51.1 (2.3)	43.9 (2)	35.0 (1.6)	54.2 (1.6)	-
Total N (mg cm^−3^)	4.7 (0.1)	2.9 (0.2)	2.8 (0.1)	4.5 (0.1)	-
Total P (µg cm^−3^)	503 (27)	180.6 (12)	280 (20)	280 (20)	-
Stems > 10 cm DBH	964	787	647	784	-
Species count	75	120	89	80	-

Means (and standard deviations) were calculated from data across each watershed reported by Turner and Dalling (2021) and Prada et al. (2017). Dashes indicate data not available

### DNA extraction and sequencing

To characterize the fungal and prokaryotic communities, DNA was extracted from 0.25 g of soil using the PowerSoil DNA Isolation Kit (MO BIO Laboratories, Carlsbad, USA). DNA extracts were submitted to the Roy J. Carver Biotechnology Center at the University of Illinois at Urbana-Champaign for PCR amplification using a Fluidigm Access Array IFC chip (see: Cronn et al. 2012), which allows for amplification of multiple primer sets simultaneously (Fluidigm, San Francisco, USA), and Illumina sequencing (Illumina, San Diego, USA). Specific details for the PCR procedures and mixtures as well as library generation can be found in Suriyavirun et al. (2019). Fungal communities were assessed via the *ITS2* gene region using ITS3 and ITS4 (White et al. 1990) primers to amplify DNA. We assessed bacterial and archaeal communities via the bacterial and archaeal *16S* rRNA genes. The samples were amplified using V4_515f forward (Parada et al. 2016) and V4_806r reverse (Apprill et al. 2015) primers. Sequence information for all primers can be found in Table S1. Both *ITS* and *16S* amplicons were sequenced via Illumina HiSeq bulk 2 × 250 bp V2. Sequence data are publicly available from the NCBI SRA database under accession number PRJNA1027860.

To assess variation in microbial communities, we generated amplicon sequence variants (ASVs) from our sequence data using the DADA2 bioinformatics pipeline (Version 1.16; Callahan et al. 2016). Sequences were quality filtered and denoised, forward and reverse reads were merged, and chimeric sequences were removed using recommended parameters for *16S* and *ITS* genes (Callahan et al. 2020). Taxonomy was assigned via the naïve Bayesian DADA2 classifier (Wang et al. 2007), using the SILVA R138.1 (Quast et al. 2012) and UNITE V9.0 (Nilsson et al. 2019) reference databases for *16S* and *ITS* taxonomic assignments, respectively. Final count numbers were relativized without rarefaction (McMurdie and Holmes 2014) via a Hellinger transformation prior to statistical analysis. Final datasets consisted of 1043 fungal, 25,817 bacterial, and 96 archaeal ASVs. More information about sequencing depth and quality can be found in supplementary Table S2 and supplementary Fig. S1. Due to being derived from the same primer sets, bacterial and archaeal communities were analyzed together. We assigned fungi to saprotrophic, pathogenic, and EM functional guilds using the FUNGuild database (Nguyen et al. 2016), classifying assignments to guilds with “probable” or “highly probable” confidence scores.

### Statistical analysis

Statistical analyses were performed in the R statistical environment (Version 4.3.2; R Core Team 2013). To understand how stand mycorrhizal type influenced microbial alpha diversity (i.e., relative ASV richness and diversity), we used Hill numbers for orders of *q* (*q* = 0, 1, 2) using the *hillR* package (Version 0.5.2; Li 2018). Hill numbers provide the “effective number of species” or “species equivalents” (MacArthur 1965; Hill 1973; Jost 2007; Chao et al. 2014): *q* = 0 is representative of richness, where all species are weighted equally; *q* = 1 is the exponential of Shannon entropy representative of diversity, where species are weighted by their proportional abundance; and *q* = 2 is equivalent to the inverse of Simpson’s index, where rare species are down-weighted. To assess differences in beta diversity patterns between stand mycorrhizal types, we used distance-based redundancy analyses (dbRDA) in the VEGAN package (Version 2.6–4; Oksanen et al. 2010) on a Jaccard distance matrix of Hellinger transformed fungal and prokaryotic ASV abundances. Stand mycorrhizal type was a fixed factor in the dbRDA, and the sampling site was partialed out via the *Condition*() arguments in the *dbrda*() function in VEGAN. We also depict these relationships visually through the dbRDA ordination without fixed or partialed effects. To assess the relative variation explained by site and stand mycorrhizal type, we used variance partitioning via the *varpart()* function in VEGAN on the Jaccard distance matrix for both fungal and bacterial/archaeal communities. To understand the correspondence between fungal and bacterial/archaeal communities, we calculated the procrustean association metric (PAM) based on the output of the *residuals*() function from procrustean correspondence between fungal and bacterial/archaeal dbRDA ordinations, *protest*() function in VEGAN. We used linear mixed models to test the effects of stand mycorrhizal type on fungal and prokaryotic Hill numbers, the relative abundance/richness of fungal functional guilds (saprotrophs, pathogens, and EM fungi), and PAM, with stand mycorrhizal type as a fixed effect and site location as a random effect. Finally, to understand whether community dissimilarity between fungal and bacteria/archaeal communities was related to the relative abundance/richness of EM, saprotrophic, or pathogenic fungi, we used a general linear model to examine the relationship between PAM and the relative abundance of fungal functional groups. We used DESeq2 (Version 3.18; Love et al. 2014) to determine bacterial, archaeal, and fungal phyla, classes, orders, families, and genera that differed significantly in abundance between stand mycorrhizal types, as this approach accounts for multiple comparisons and overdispersion among taxonomic count numbers. Significance for all statistical test was assessed as *p* < 0.05.

## Results

**Fig. 1:**
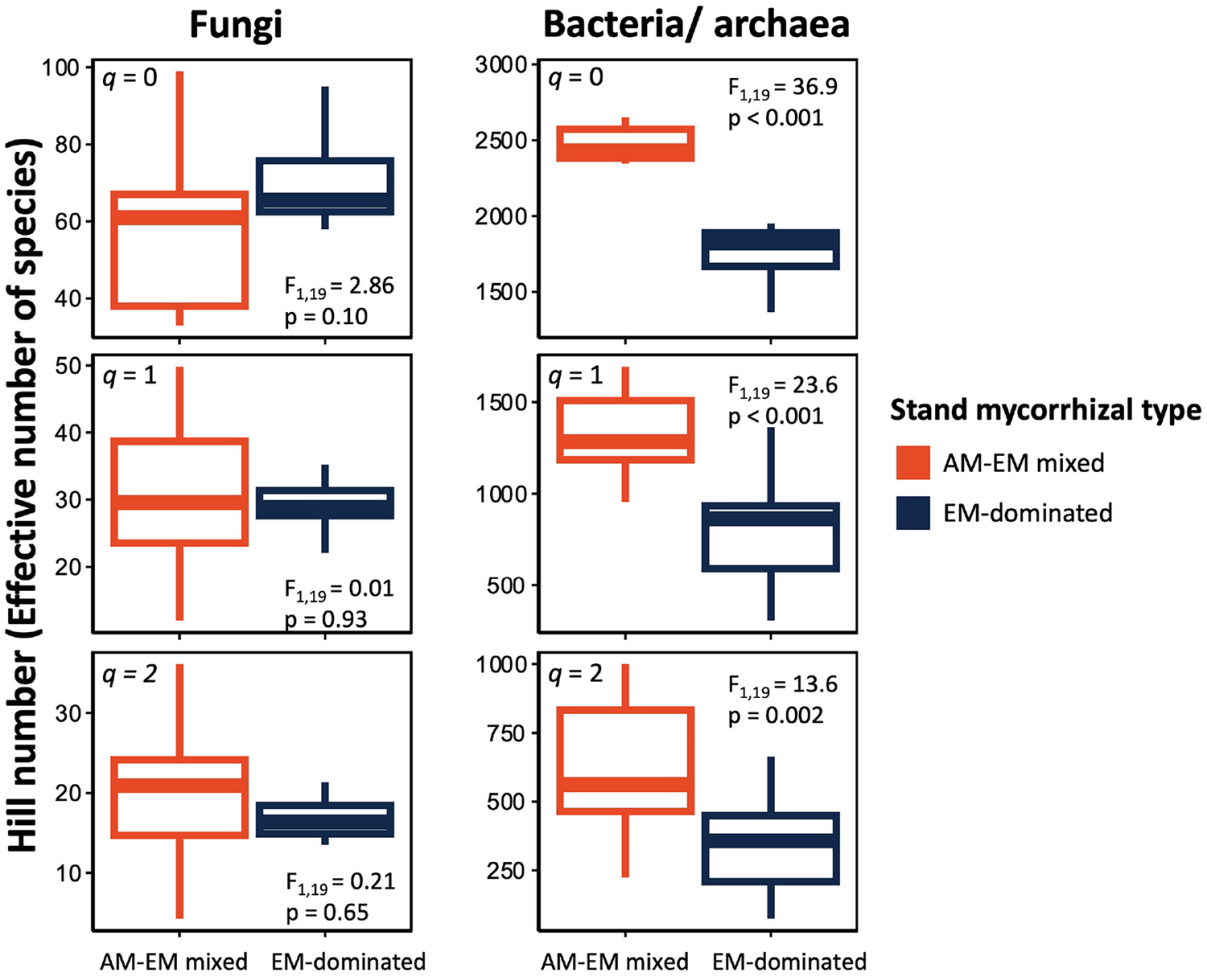
Mean (25th, 75th percentile, lowest and highest values observed) fungal and bacterial alpha diversity between ectomycorrhizal (EM) and mixed arbuscular mycorrhizal (AM)-EM stand types using Hill numbers depicting effective number of species with all ASVs weighted equally (*q* = 0), ASVs weighted based on their proportional abundance (*q* = 1), and rare ASVs down-weighted (*q* = 2)

Ectomycorrhizal-dominated stands had significantly lower alpha diversity (Hill numbers) for bacterial/archaeal communities at all orders of *q*, with 27% fewer effective number of species at *q* = 0, 40% fewer effective number of species at *q* = 1, and 46% fewer effective number of species at *q* = 2 (*p* < 0.05, Fig. 1). In contrast, we found no significant difference between EM-dominated and mixed AM-EM stands for fungal Hill numbers at any order of *q* (Fig. 1).

**Fig. 2:**
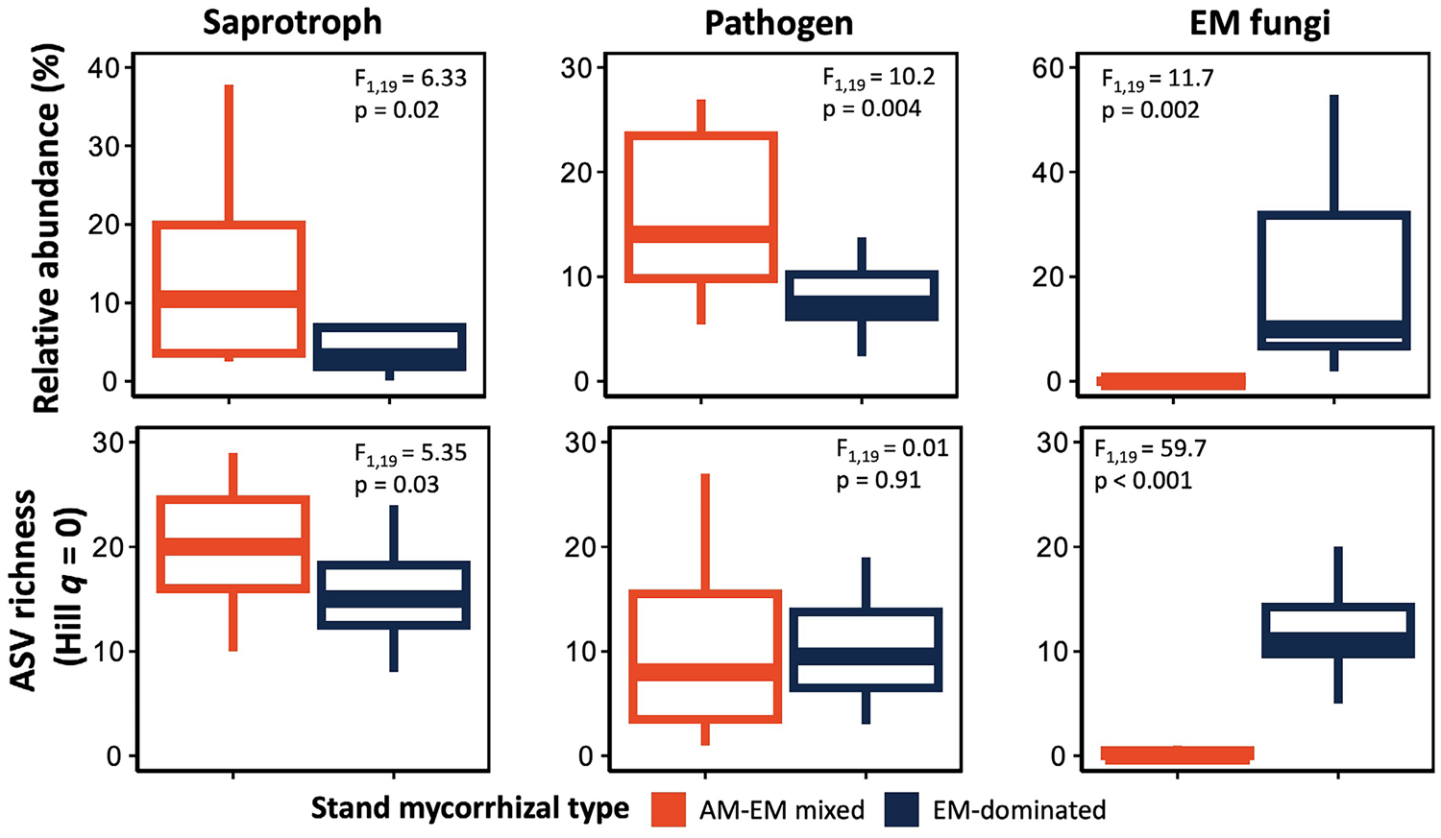
Mean (25th, 75th percentile, lowest and highest values observed) of the relative abundance (%) and ASV richness (Hill *q* = 0) of fungal saprotrophic, pathogenic, and ectomycorrhizal (EM) fungal functional guilds between EM and mixed arbuscular mycorrhizal (AM)-EM stand mycorrhizal types

The relative abundances and ASV richness of fungal functional guilds differed significantly between stand mycorrhizal types. The relative abundance of EM fungal ASVs was significantly greater in EM-dominated stands (average 180%) relative to mixed AM-EM stands (*F*_1,19_ = 11.75, *p* = 0.002; Fig. 2). Richness of EM fungal ASVs was also significantly greater (average 170%) relative to mixed AM-EM stands (*F*_1,19_ = 59.7, *p* < 0.001; Fig. 2). On average, saprotrophic fungal ASV relative abundance was 56% lower in EM-dominated than mixed AM-EM stands (*F*_1,19_ = 6.33, *p* = 0.02), with ASV richness of fungal saprotrophs decreasing 27.5% from EM-dominated to AM-EM mixed stands (*F*_1,19_ = 5.35, *p* = 0.03). Pathogenic fungal ASV relative abundance was 50% lower in EM-dominated stands than AM-EM mixed stands (*F*_1,19_ = 10.2, *p* = 0.004; Fig. 2); however, pathogenic ASV richness was not different between stand mycorrhizal types. We did not detect significant correlations among the ASV relative abundances of saprotrophic, pathogenic, and EM functional guilds (*p* > 0.05). We also did not find a significant difference between stand mycorrhizal types in the relative abundance of ASVs not assigned to saprotrophic, pathogenic, or EM functional guilds (average 68.2%, *p* > 0.05; Fig. S2).

**Fig. 3:**
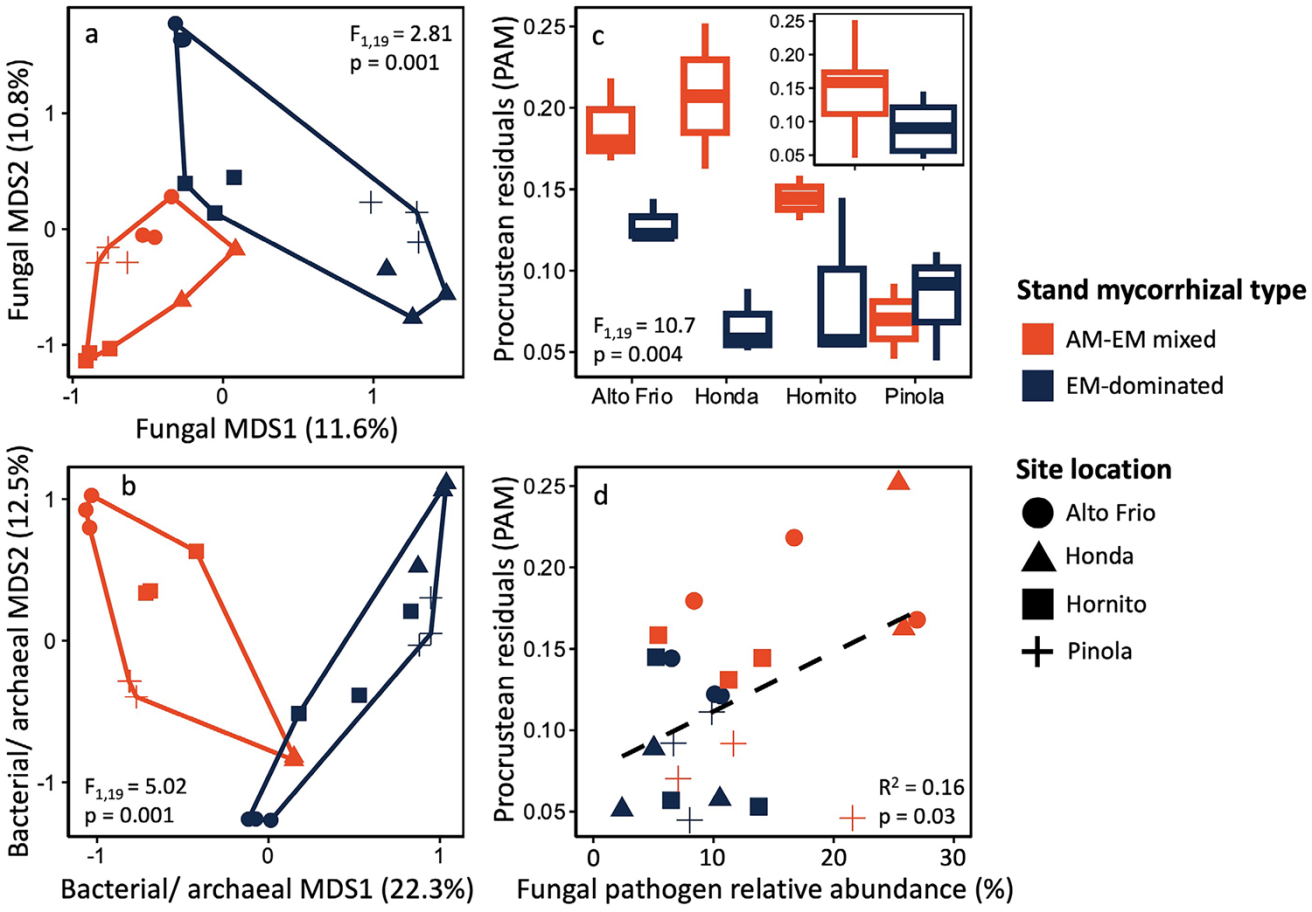
Fungal (**a**) and bacterial (**b**) community ordinations with axes indicating the proportion of community variation explained by the first and second multidimensional dimension scaling (MDS). Procrustean residuals between fungal and bacterial community ordinations with site-specific differences shown in the panel and overall difference between stand mycorrhizal type shown in insert (**c**). Relationship between procrustean residuals and fungal pathogen relative abundance (**d**)

Fungal and bacterial/archaeal communities both differed significantly between stand mycorrhizal types, but the ways in which these communities differed, and the potential drivers of these differences were not the same for fungi and prokaryotes. Stand mycorrhizal type explained 7.71% of the variation in fungal community composition among samples (dbRDA: *F*_1,19_ = 2.81, *p* = 0.001), while site explained 13.1% of variations. Fungal communities in EM-dominated stands had significantly greater dispersion than those in mixed AM-EM stands (betadisper: *F*_1,21_ = 5.28, *p* = 0.03; Fig. 3a). Stand mycorrhizal type explained 15.3% of the variation in bacterial/archaeal community composition (*F*_1,19_ = 5.02, *p* = 0.001), while site explained 14.1% of the variation. We did not detect significant stand mycorrhizal type differences in bacterial/archaeal community homogeneity (Fig. 3b).

There was a significant Procrustean rotational similarity correlation between fungal and bacterial/archaeal ordinations (*R*^2^ = 0.78, *p* = 0.001). However, Procrustean residuals (PAM) were significantly lower (i.e., greater resemblance) in EM-dominated than mixed AM-EM stands (Fig. 3c). Further, there was a significant positive relationship between PAM and fungal pathogen relative abundance (*R*^2^ = 0.16, *p* = 0.03; Fig. 3d). We did not detect a similar relationship between PAM and the relative abundance of saprotrophic or EM fungi (Fig. S3; *p* > 0.05).

**Fig. 4:**
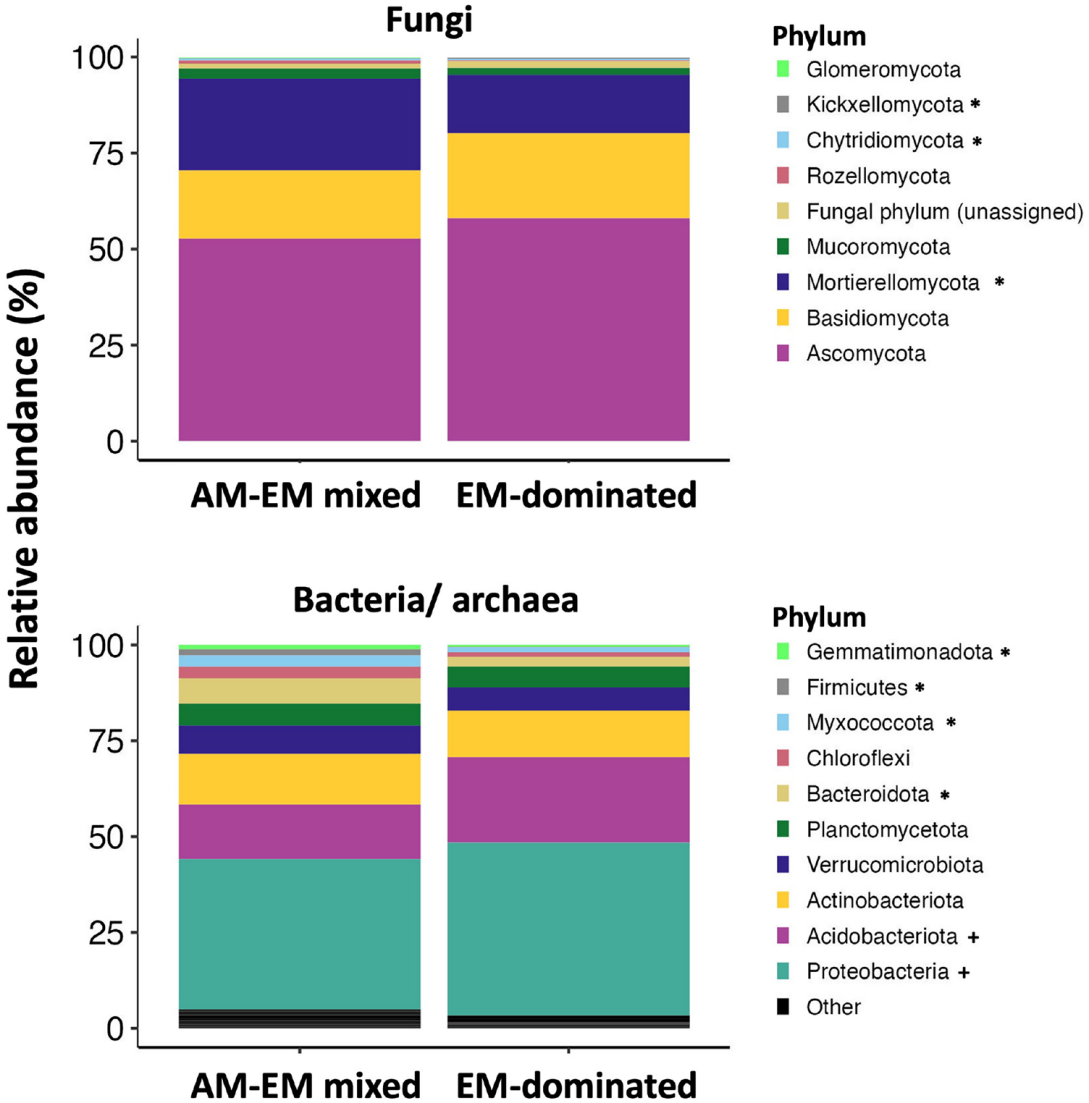
Relative abundance of the top 10 fungal and bacterial/archaeal phyla in ectomycorrhizal (EM-dominated) and arbuscular mycorrhizal-EM (AM-EM mixed) tree stands. Adjacent to phylum names, * indicates a group significantly more abundant in AM-EM mixed plots while + indicates a group significantly more abundant in EM-dominated plots

The dominance of EM-associated trees was significantly correlated with the abundance of fungal and bacterial/archaeal groups at various phylogenetic levels, with some taxonomic groups exhibiting increased and others decreased relative abundances in mixed AM-EM compared to EM-dominated stands. We found that 3 fungal phyla (Mortierellomycota, Kickxellomycota, and Chytridiomycota) were less abundant in EM-dominated than mixed AM-EM stands (Table S3). For fungi overall, 5 classes, 6 orders, 12 families, and 18 genera were less abundant in EM-dominated stands, while three classes, 4 orders, 8 families, and 9 genera were more abundant in these stands (Table S3). For prokaryotes, 10 among the 16 phyla that responded to stand mycorrhizal type were less abundant in EM-dominated stands (e.g., Bacteroidota, Myxococcota, Firmicutes, Gemmatimonadta, Methylomirabilota, Latescibacterota, Nitrospirota, MBNT15, Entotheonellaeota, and SAR324), while Proteobacteria, Acidobacteriota, Planctomyceteota, RCP2-54, WPS-2, and Armatimonadota were more abundant in EM-dominated stands (Table S4, Fig. 4). For prokaryotes overall, 29 classes, 86 orders, 153 families, and 227 genera were less abundant in EM-dominated stands (Table S4). Comparatively, far fewer prokaryotic groups were more abundant in EM-dominated stands, with 8 classes, 19 orders, 20 families, and 32 genera falling into this category (Table S4). However, these groups often represented the most abundant bacterial/archaeal groups overall, as shown in the graphical depictions of the relative abundance of the top 10 bacterial groups at these phylogenetic levels (Figs. S4–S7).

## Discussion

We found a strong relationship between EM dominance and bulk soil microbiome composition in diverse neotropical montane forests. The relative abundance of EM-associating tree species in tropical forests is associated with significant shifts in soil properties (Barceló et al. 2022), yet it remains unclear how these shifts influence soil microbial composition. In support of our hypothesis, we found clear differentiation in soil microbial communities between stand mycorrhizal types across a range of forests differing in parent material and soil chemical properties, with stand mycorrhizal type explaining slightly greater variation in prokaryotic communities than site location. In relation to our predictions, (1) we demonstrate that EM-dominated forest stands maintain a less diverse prokaryotic microbiome than mixed AM-EM stands, (2) EM-dominated stands have lower relative abundance of fungal saprotrophs and pathogens than surrounding AM-EM mixed stands with taxonomic shifts of prokaryotes aligning with expected functional shifts for SOM cycling, and (3) correspondence between fungal and prokaryotic communities was greater in EM-dominated stands than in AM-EM mixed stands, but this did not correlate with the relative abundance of EM fungi. Our findings suggest that mycorrhizal fungi may affect the composition of non-mycorrhizal communities either directly or indirectly, through mycorrhizal effects on plant communities (Liang et al. 2020) and soil nutrient economies (Corrales et al. 2016b).

Ectomycorrhizal tree and fungal microbiome recruitment may have influenced the lower prokaryotic diversity we observed in EM-dominated than in mixed AM-EM stands. Tree species can independently recruit distinct bacterial communities (Oh et al. 2012). The EM-dominated stands we studied have a much lower overall tree species diversity than surrounding AM-EM mixed stands (Prada et al. 2017), potentially explaining higher prokaryotic community diversity in these stands. At our study site, EM leaf litter is not necessarily lower quality than AM leaf litter (Seyfried et al. 2021b). However, leaf litter in EM-dominated stands may be more chemically homogenous than leaf litter in mixed species AM stands where a high diversity of AM tree species contributes litter ranging widely in chemical quality to the forest floor (Seyfried et al. 2021b). Chemical heterogeneity could increase overall niche breath for microorganisms to support a highly diverse bacterial/archaeal community in AM-EM mixed stands. Additionally, mycorrhizal fungi may affect prokaryote communities directly by recruiting specific hyphosphere bacterial communities (Liu et al. 2018; Heděnec et al. 2020; Zhang et al. 2022). Specifically, AM fungi can support bacterial growth to facilitate inorganic nutrient transformations (Wang et al. 2023), while EM fungi may compete with free-living decomposers for organic nutrients (Fernandez and Kennedy 2016). These mycorrhizal hyphosphere responses also may be driven by differing patterns of belowground C allocation and root/fungal exudation between AM- and EM-associating tree and fungal species (Xu et al. 2023). Mycorrhizae and prokaryotic communities show strong patterns of interactions that may be driven by above- and belowground functional differences between AM and EM guilds.

Shifts in alpha diversity in response to stand mycorrhizal type also corresponded to functional changes among prokaryotic taxa. The influence of EM-dominated versus AM-EM mixed stand type on microbiome recruitment could have driven the lower relative abundance of bacterial/archaeal groups associated with inorganic nutrient transformation processes, such as Nitrospirota (Myrold 2021), in EM-dominated stands relative to AM-EM mixed stands. Microbiota beneficial to AM fungi could have been inhibited either directly, or indirectly through EM effects on inorganic N availability (Phillips et al. 2013; Corrales et al. 2016b). Further, there may be a relationship between these changes to functionally important taxa and EM-associated C dynamics. Ectomycorrhizal-dominated stands favored bacterial groups associated with diminished C mineralization activities, with greater Acidobacteriota and lower Bacteroidota relative abundance (Fierer et al. 2007) than in mixed AM-EM stands. These differences in the taxonomic composition of prokaryotic communities suggest that there may also be lower overall rates of C cycling and SOM decomposition in AM-EM mixed stands than in EM-dominated stands. Alternatively, *O. mexicana* may produce allelochemicals, as has been observed for other members of Juglandaceae (Jose and Gillespie 1998), which could be responsible for directly inhibiting specific soil microbiota (Revillini et al. 2023). This mechanism could potentially explain negative plant-soil feedbacks associated with *O. mexicana* legacy, although further investigation is necessary to determine the presence and identity of any possible allelopathic chemicals this plant could produce. Overall, suppression of C and N cycling by EM fungi and low tree diversity resulting in chemically homogenous root and leaf litter inputs in EM-dominated stands may promote specific microbiome assembly, potentially providing EM trees a competitive advantage.

Overall fungal community beta diversity may have been driven by mycorrhizal interactions with their environment. We did not find a decrease in fungal alpha diversity in EM-dominated stands relative to mixed AM-EM stands as has been reported in temperate forests (Eagar et al. 2021). Rather, fungal communities were more heterogeneous among EM-dominated stands than among mixed AM-EM stands. Greater fungal community heterogeneity across EM-dominated stands could have been driven by soil pH and fertility which varied across our four watersheds and can select for functionally distinct EM fungal communities (Corrales et al. 2016a). Specifically, low pH and fertility in Honda may select EM fungi that have a great capacity to alter C and N cycling through organic N uptake and that contribute abundant, low-quality fungal biomass to SOM pools (Seyfried et al. 2022). In contrast, relatively high soil pH and fertility in Alto Frio may select EM fungi which exclusively take up inorganic N and contribute limited, high-quality biomass to SOM pools (Seyfried et al. 2021a). These selective forces on EM fungi may represent a disproportionate influence on the overall fungal community based on the high relative abundance of EM fungi in these EM-dominated stands. In mixed AM-EM stands, environmental filtering for functionally robust, less specialized fungal communities may have driven greater homogeneity across sites despite differences in underlying soil pH and fertility (Kivlin et al. 2018). Shifts in fungal communities between stand mycorrhizal types were also correlated with higher relative abundance of EM fungi and lower relative abundance of fungal pathogens and saprotrophs in EM-dominated stands than in the AM-EM mixed stands. Interactions among fungal guilds may help promote positive plant-soil feedbacks associated with EM mutualisms (Bennett et al. 2017) by creating favorable microbiomes (i.e., lower potential pathogen loads and decomposer abundance) in EM-dominated forest stands. The functional composition of soil fungal communities in EM-dominated forest stands may partially drive the effects of EM trees on ecosystem function (McGuire et al. 2010) and be influenced by underlying soil pH and fertility.

The relationships between fungal and prokaryotic communities may highlight tradeoffs in belowground dynamics for different stand mycorrhizal types. Fungal and prokaryotic communities were more closely associated in EM-dominated than in mixed AM-EM stands. However, the divergence in this relationship (Procrustean residuals) increased with the relative abundance of fungal pathogens. Tradeoffs between defense, growth, nutrient acquisition, and mutualist collaboration in the development and morphology of root structures are an important component of plant development (Ravanbakhsh et al. 2019; Bergmann et al. 2020; Monson et al. 2022). For example, in tropical forests, the species most proficient at acquiring soil phosphorus are also the most vulnerable to pathogens (Laliberté et al. 2015; Lambers et al. 2018), with this tradeoff hypothesized to potentially help to maintain high plant diversity in these systems. Divergences between fungal and prokaryotic communities could be representative of tradeoffs made by plants to alter resource allocation for microbiome assembly in favor of pathogen protection. While this argument is largely speculative, the relationships we present here provide further support for microbial communities (or the capacity to manipulate them) as an extended plant root trait, which may be influenced by the surrounding environmental context (Freschet et al. 2021).

## Conclusion

In a tropical montane forest, we demonstrate that EM-dominated stands are characterized by decreased prokaryotic diversity and different relative abundances of several important microbial functional groups compared to surrounding diverse mixed AM-EM forest. Differences in microbial communities between stand mycorrhizal types could be driven by overall differences in plant communities or microbiome assembly but likely contribute to broad EM-associated ecosystem effects. The relationships among different constituents of the soil microbiome could be an important extension of mycorrhizal function in response to the surrounding environment. Distinct soil microbiomes in EM-dominated versus mixed AM-EM stands may contribute to the effects of EM relationships on plant health or ecosystem function via changes to the relative abundance of fungal pathogens and saprotrophs. Overall, we found that the effects of mycorrhizal associations extend beyond the plant-fungal partnership into the broader soil microbiome and are especially pronounced for soil bacterial/archaeal communities.

### Supplementary Information

Below is the link to the electronic supplementary material.

Supplementary file1 (DOCX 1741 KB)

Supplementary file2 (CSV 6 KB)

Supplementary file3 (CSV 56 KB)
