# Supplementary material for: Soil microbial community response to ectomycorrhizal dominance in diverse neotropical montane forests
Source: Mycorrhiza. 2024 Jan 6;34(1-2):95–105. doi: 10.1007/s00572-023-01134-4 (PMC10998807; doi:10.1007/s00572-023-01134-4)

| **Target gene** | **PCR primers** | **Primer sequence (5’→3’)** | **Reference** |
| --- | --- | --- | --- |
| **Fungal ITS forward** | ITS3 | GCATCGATGAAGAACGCAGC | White et al. (1990) |
| **Fungal ITS reverse** | ITS4 | TCCTCCGCTTATTGATATGC | White et al. (1990) |
| **Bacterial and Archaeal 16S forward** | V4_515F | GTGYCAGCMGCCGCGGTAA | Parada et al. (2016) |
| **Bacterial and Archaeal 16S reverse** | V4_806R | GGACTACNVGGGTWTCTAAT | Apprill et al. (2015) |

Table S1. Target genes, PCR primer names, primer sequences, and references for genes sequences in fungal and bacterial/ archaeal community analysis.

| Target gene | | Raw read # | Quality filtered | % reads retained |
| --- | --- | --- | --- | --- |
| ITS | Mean | 24277 | 22895 | 94 |
|  | Standard Error | 871 | 814 | <1.0 |
| 16S | Mean | 111107 | 68398 | 62 |
|  | Standard Error | 2284 | 1706 | 1.0 |

Table S2. Raw read count, number of reads after bioinformatic processing, and % read retained for *ITS* and *16S* amplicon sequence data.

Supplementary Figure S1. Species accumulation curves for fungal and bacterial/ archaeal sequence counts in final data used for analysis.
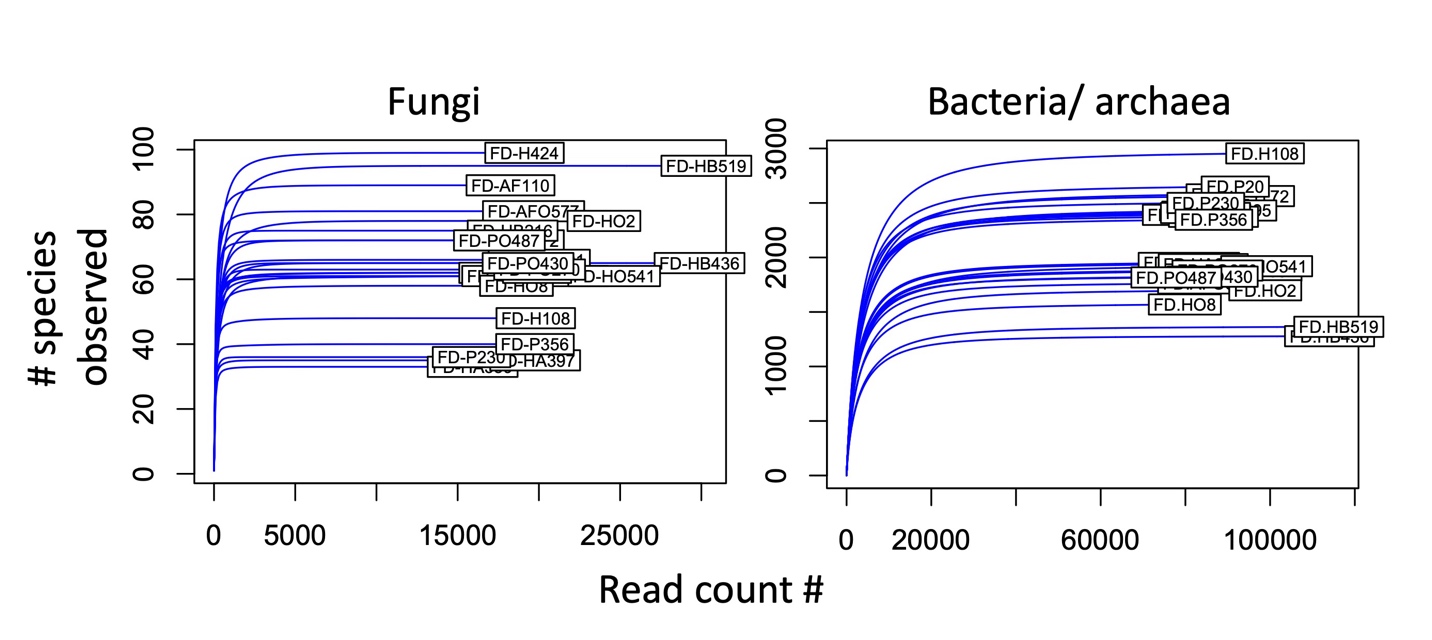


Supplementary Figure S2. Guild stuff


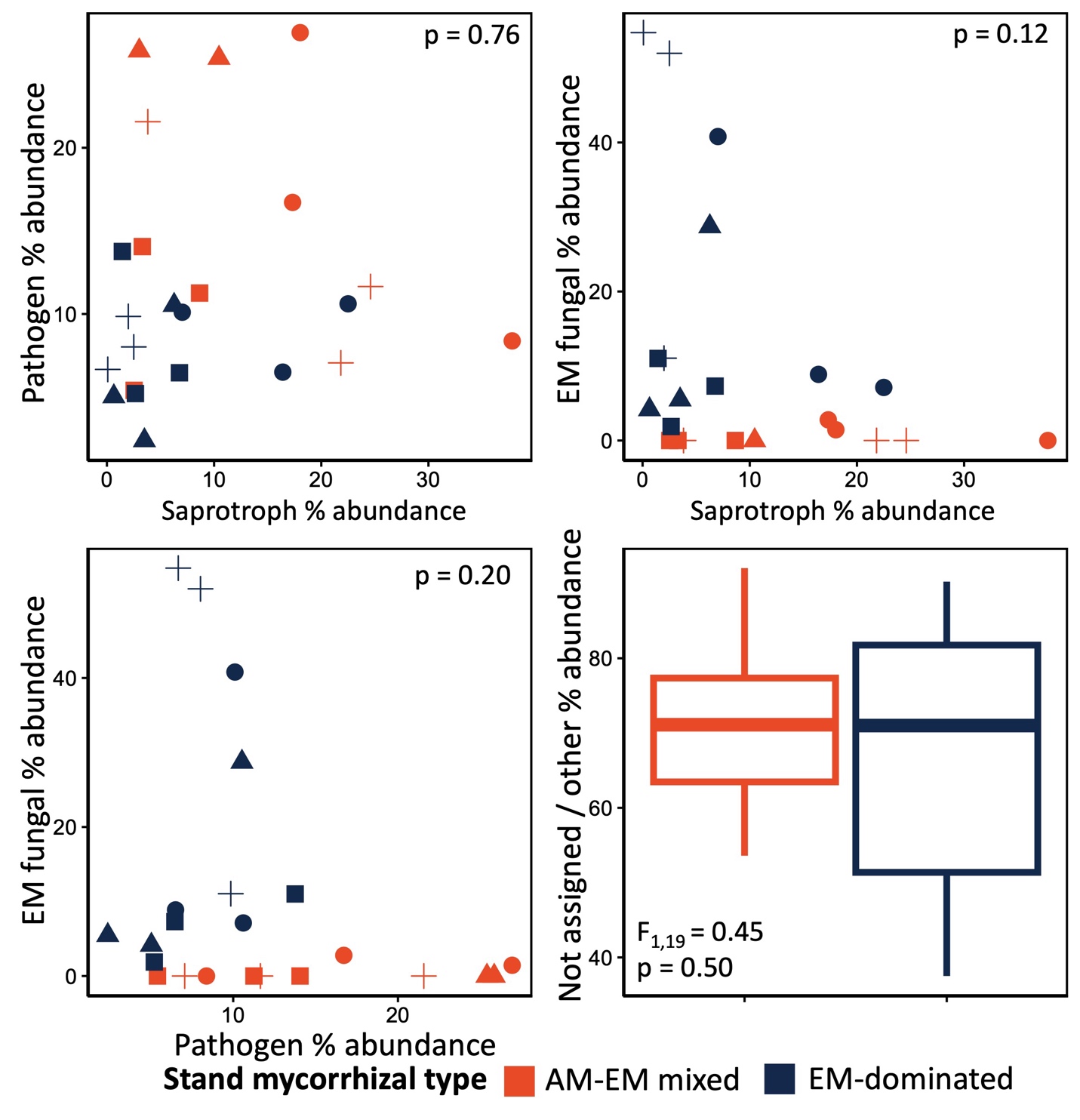


Supplementary Figure S3. Correlations between saprotrophic, pathogenic, and EM fungal guilds with Procrustean residuals (PAM).


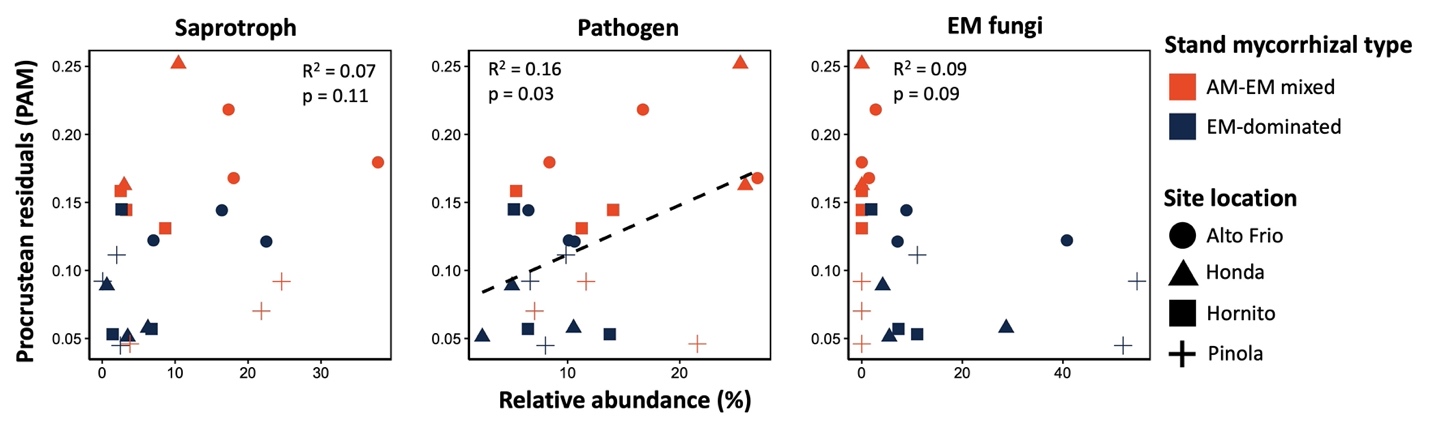


Supplementary Figure S4. Relative abundance of the top 10 fungal and bacterial/ archaeal classes in AM-EM mixed and EM-dominated tree stands. In label names, * indicates a group more abundant in AM-EM mixed plots while + indicates a group more abundant in EM-dominated plots.


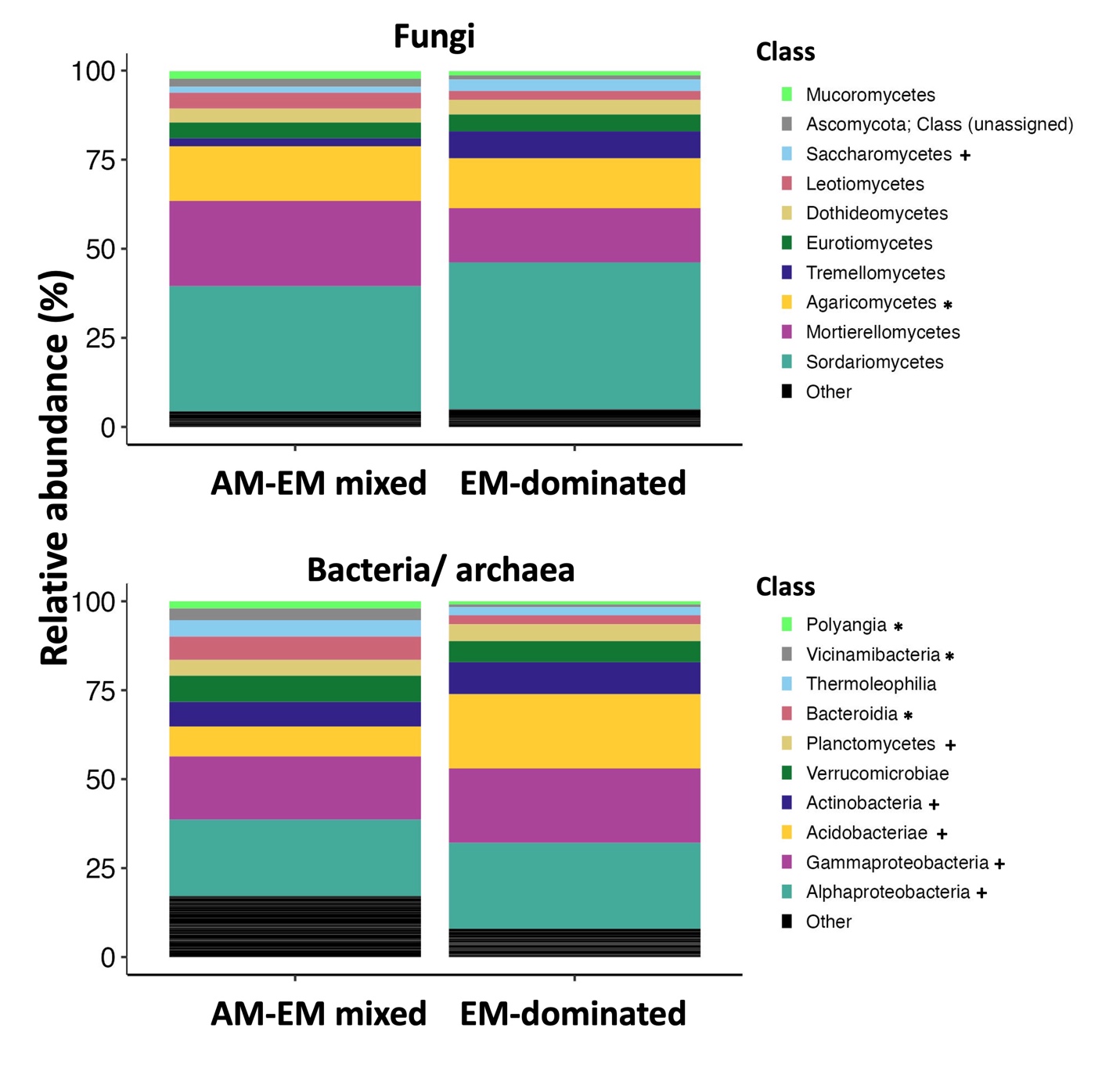


Supplementary Figure S5. Relative abundance of the top 10 fungal and bacterial/ archaeal orders in AM-EM mixed and EM-dominated tree stands. In label names, * indicates a group more abundant in AM-EM mixed plots while + indicates a group more abundant in EM-dominated plots.


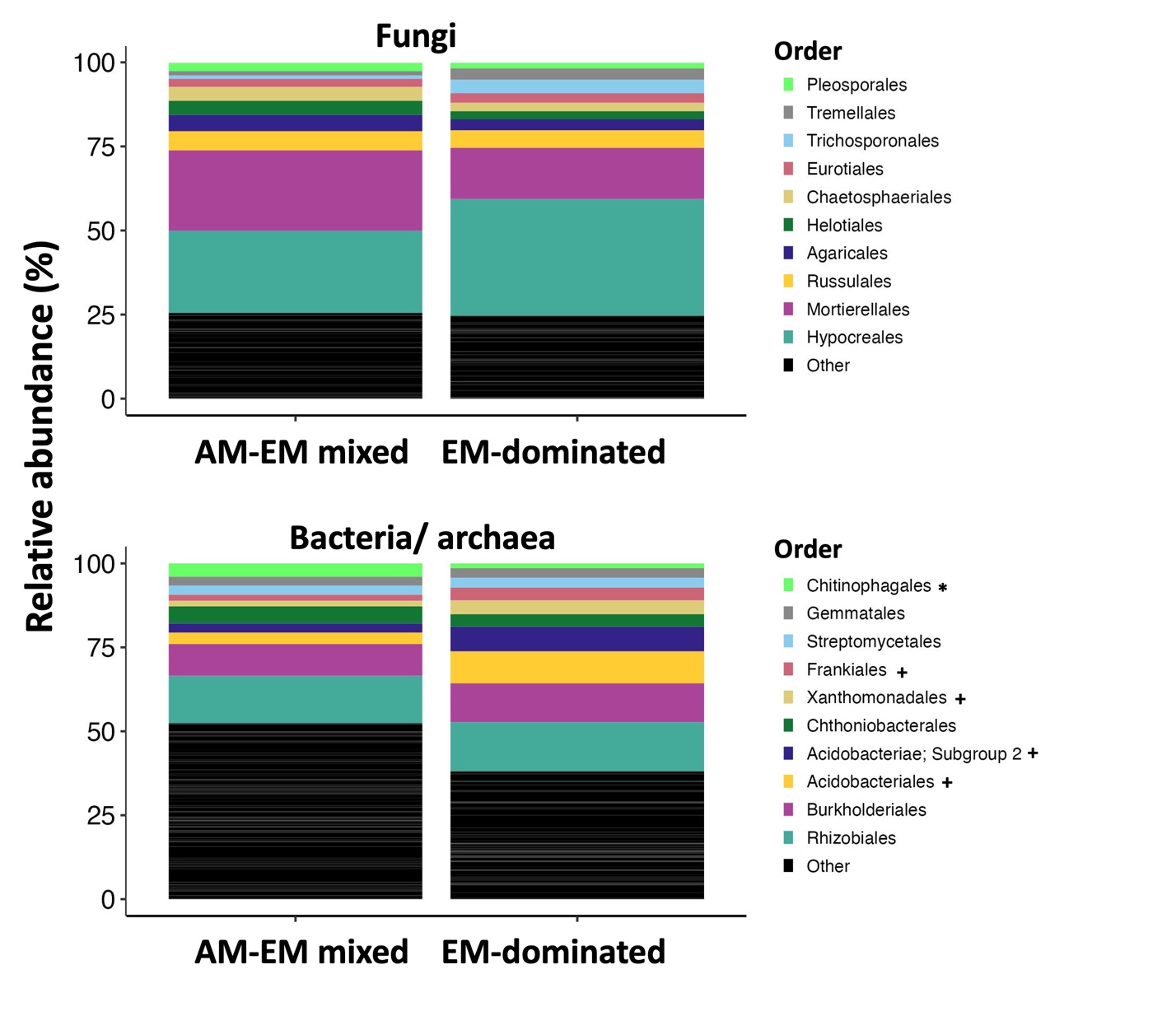


Supplementary Figure S6. Relative abundance of the top 10 fungal and bacterial/ archaeal families in AM-EM mixed and EM-dominated tree stands. In label names, * indicates a group more abundant in AM-EM mixed plots while + indicates a group more abundant in EM-dominated plots.


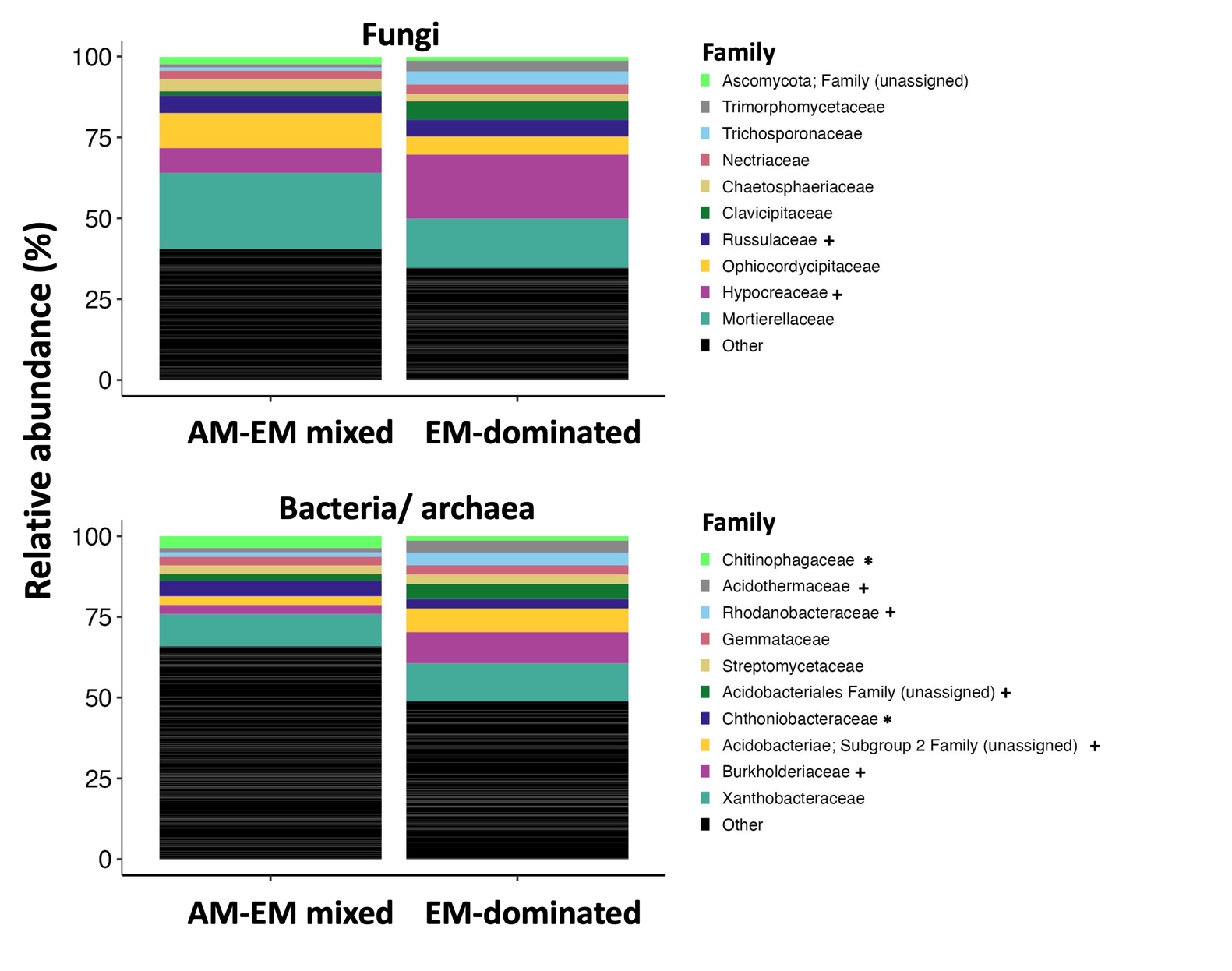


Supplementary Figure S7. Relative abundance of the top 10 fungal and bacterial/ archaeal genera in AM-EM mixed and EM-dominated tree stands. In label names, * indicates a group more abundant in AM-EM mixed plots while + indicates a group more abundant in EM-dominated plots.


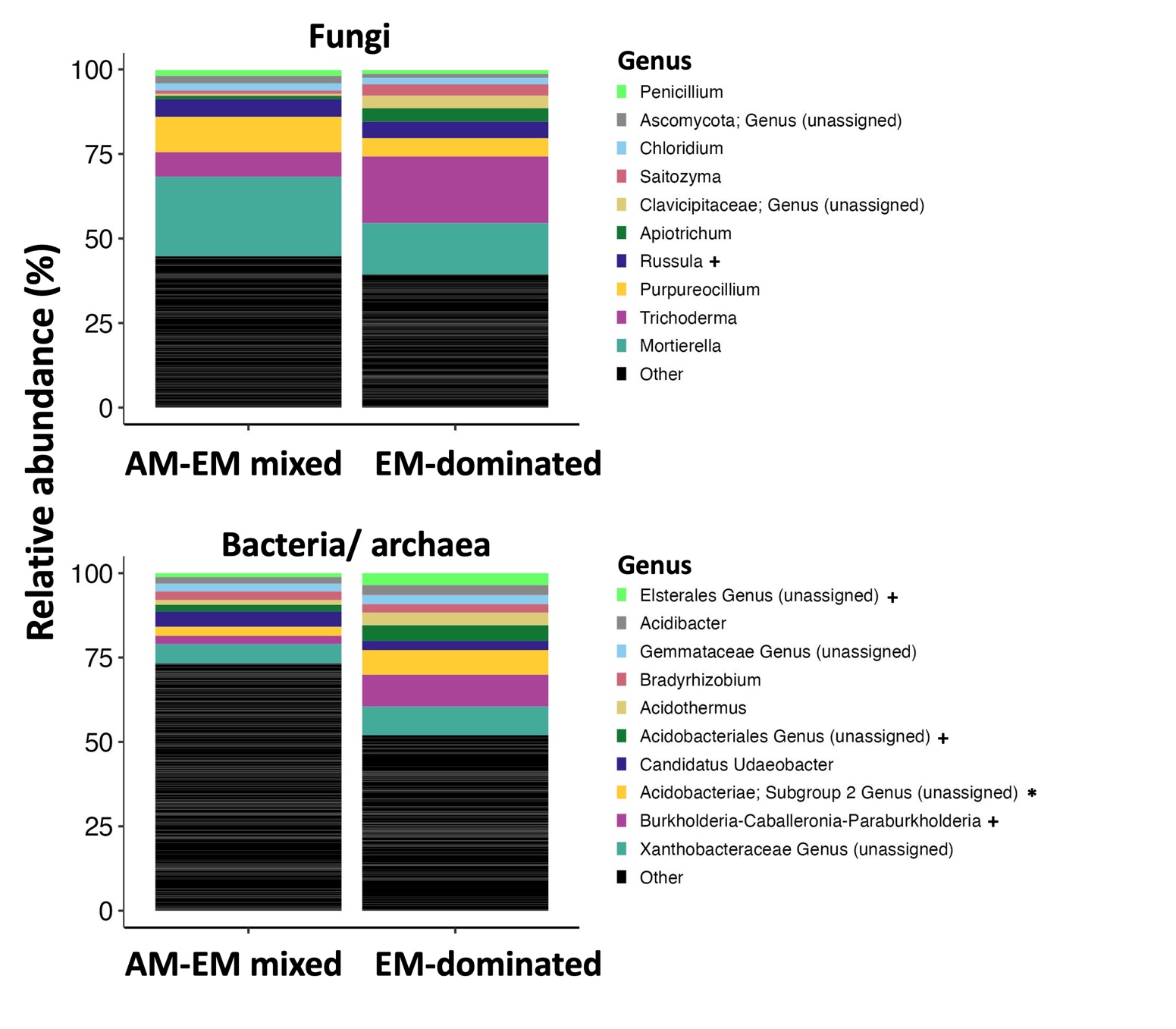

Supplement: Supplementary file 1 — Supplementary file1 (DOCX 1741 KB) [file 572_2023_1134_MOESM1_ESM.docx]
